# UHMK1 Promotes Prostate Cancer Progression through a Positive Feedback Loop with MTHFD2

**DOI:** 10.32604/or.2025.065119

**Published:** 2025-08-28

**Authors:** Chi Zhang, Xi Huang, Cheng Hu, Bowen Tang, Jianjie Wu, Zhuolun Sun, Weian Zhu, Xiangfu Zhou, Hengjun Xiao, Hua Wang

**Affiliations:** 1 Department of Urology, The Third Affiliated Hospital, Sun Yat-sen University, Guangzhou, 510630, China; 2 Department of Ultrasound, Sun Yat-sen Memorial Hospital, Sun Yat-sen University, Guangzhou, 510120, China

**Keywords:** Cancer progression, prostate cancer (PCa), positive feedback loop, methylenetetrahydrofolate dehydrogenase 2 (MTHFD2), U2AF Homology Motif Kinase 1 (UHMK1)

## Abstract

**Background:**

U2AF homology motif kinase 1 (UHMK1) has been associated with RNA processing and protein phosphorylation, thereby influencing tumor progression. The study aimed to explore its regulatory mechanisms and biological functions in human prostate cancer (PCa).

**Methods:**

In this study, we systematically evaluated the expression and prognostic significance of UHMK1 in public databases, followed by validation through immunohistochemistry (IHC) in PCa specimens. Both gain-of-function and loss-of-function experiments were conducted to elucidate the role of UHMK1 *in vitro* and *in vivo*. Additionally, a series of molecular and biochemical assays were performed to investigate the regulatory mechanisms underlying UHMK1 activity.

**Results:**

Our findings revealed that UHMK1 expression was significantly upregulated in PCa tissues and correlated with poor patient prognosis, as demonstrated by analysis of public datasets and confirmed by immunohistochemical staining. Functional studies showed that UHMK1 depletion suppressed tumor cell proliferation and metastasis, while its overexpression promoted these processes. Mechanistically, we identified that UHMK1 phosphorylates nuclear receptor coactivator 3 (NCOA3), which subsequently activates activating transcription factor 4 (ATF4) to upregulate methylenetetrahydrofolate dehydrogenase 2 (MTHFD2) transcription. Interestingly, MTHFD2 was found to reciprocally enhance UHMK1 expression, establishing a positive feedback loop.

**Conclusions:**

In conclusion, our data suggest that the UHMK1-MTHFD2 axis forms a positive feedback loop that drives PCa progression. Targeting this loop represents a promising therapeutic strategy for restraining prostate cancer development and progression.

## 1 Introduction

Prostate cancer (PCa) is one of the most prevalent cancers among men worldwide [1]. Approximately one-third of patients are diagnosed with advanced PCa at their first visit, rendering them ineligible for radical therapy [2–4]. Androgen deprivation therapy (ADT) remains the cornerstone of treatment at this stage [5]. While initially effective, ADT often becomes ineffective once PCa progresses to castration-resistant prostate cancer (CRPC) [6]. Currently, limited effectiveness poses a significant clinical challenge in treating CRPC. Further research into PCa pathogenesis and molecular mechanisms may help aid in identifying novel treatment strategies for managing advanced PCa in the future.

U2AF homology motif kinase 1 (UHMK1) is a distinctive serine/threonine kinase initially identified as a regulator of the cell cycle [7,8]. UHMK1 has been shown to interact with various proteins, including splicing factor 1 (SF1) and α-amino-3-hydroxy-5-methyl-4-isoxazolepropionic acid (AMPA), suggesting roles in pre-mRNA splicing and other biological processes [9–11]. For example, elevated UHMK1 expression correlates with poor prognosis, while silencing UHMK1 inhibits tumor proliferation and xenograft growth in pancreatic ductal adenocarcinoma [12]. Moreover, in melanoma, UHMK1 inhibits tumor cell death by reprogramming mitochondrial metabolism [13]. However, the expression of UHMK1 in PCa is not clear, let alone its biological functions.

Methylenetetrahydrofolate dehydrogenase 2 (MTHFD2) is widely recognized as a key enzyme involved in mitochondrial folate one-carbon metabolism [14]. MTHFD2 is considered bifunctional due to both its metabolic roles and nonenzymatic activities [15]. It has been shown to contribute to cancer proliferation and is thought to play a significant role in tumor development and progression [16–18]. By analyzing next-generation sequencing (NGS) data, we identified MTHFD2 as a potential downstream target of UHMK1. However, it remains to be tested whether MTHFD2 plays a specific role in the progression of PCa.

In this study, our aim was to investigate the expression of UHMK1 in prostate cancer, as well as its biological functions and regulatory mechanisms during prostate progression. Additionally, we further explored the interaction mechanism between UHMK1 and MTHFD2.

## 2 Materials and Methods

### 2.1 Genotype-Tissue Expression (GTEx) and the Cancer Genome Atlas (TCGA) Data Acquisition

RNA sequencing (RNA-seq) data and associated clinical information for normal prostate and prostate cancer (PCa) tissues were sourced from the GTEx and TCGA databases (https://gtexportal.org/home/; https://portal.gdc.cancer.gov) (accessed on 8 July 2025). Patients with incomplete clinical data or unavailable survival data were excluded from this analysis. Samples were stratified according to their Gleason scores (GS) and N stages [19] for subsequent analysis.

### 2.2 Clinical Specimens and Survival Data

Benign prostatic hyperplasia (BPH) specimens (n = 6), PCa tissues (n = 112) and matched adjacent non-tumor normal tissues (GS < 7, n = 6; GS = 7, n = 6; GS > 7, n = 6) were obtained from patients treated at the Third Affiliated Hospital of Sun Yat-sen University between 2016 and 2020. Clinicopathological data and postoperative follow-up information were collected from 112 patients with prostate cancer. The experimental protocols involving human specimens in this study complied with the Declaration of Helsinki and were approved by the Medical Ethics Board of the Third Affiliated Hospital of Sun Yat-sen University (II2023-059-01), and written informed consent was obtained from each subject.

### 2.3 Immunohistochemical (IHC) Staining

BPH and PCa specimens underwent fixation and processing for IHC following established protocols [20]. Sections were incubated with UHMK1 polyclonal primary antibody (1:100; Proteintech, 11624-1-AP, Chicago, IL, USA). Imaging was utilized with an Olympus IX71 inverted microscope (Olympus Corporation, Tokyo, Japan). Staining intensity was graded: 0 (negative), 1 (weak), 2 (moderate), 3 (strong). The percentage of stained cells was categorized: 0 (0%), 1 (<10%), 2 (11%–50%), 3 (51%–80%), 4 (>80%). The immunoreactive score (IRS) was calculated as the product of intensity and percentage scores (intensity grade × percentage score).

### 2.4 Western Blot

Proteins were extracted from tissues or whole cells using a Whole Cell Lysis Assay (KeyGenBioTECH, KGP250, Nanjing, China). Nuclear and cytoplasmic fractions were isolated with a Nuclear/Cytoplasmic Protein Extraction Kit (KeyGenBioTECH, KGP150), adhering to the supplier’s guidelines. Western blot analysis was performed using established methods [21]. Primary antibody specifics are listed in Table S1. Enhanced Chemiluminescence (ECL; Affinity, KF8005, Melbourne, VIC, Australia) enabled band detection, and ImageJ software (National Institutes of Health (NIH), Bethesda, MD, USA) was utilized for density quantification.

### 2.5 Reverse Transcription Quantitative Polymerase Chain Reaction (RT-qPCR)

Total RNA was extracted from tissues or whole cells using the HP Total RNA Kit (Omega, R6812-02, Norcross, GA, USA) and reverse-transcribed with the PrimeScript RT Master Mix (Takara, RR036A, Otsu-shi, Japan). RT-qPCR was performed on a LightCycler 480 system (Roche, Indianapolis, IN, USA) using a TB Green Premix Ex Taq II (TliRNase HPlus) kit (Takara, RR820A, Otsu-shi, Japan). Relative mRNA expression was calculated via the 2^−ΔΔCt^ method, with GAPDH as the internal control. Primer sequences are listed in Table S2.

### 2.6 Cell Culture and Drug Treatments

RWPE-1, LNCaP, 22RV1, DU145, PC3, and HEK-293T cells were obtained from the American Type Culture Collection (ATCC; Manassas, VA, USA). All the cell lines were subjected to Short Tandem Repeat (STR) authentication and confirmed to be mycoplasma-free. RWPE-1 cells were cultured in keratinocyte-serum-free medium (SFM; Gibco, 17005042, Grand Island, NY, USA), while LNCaP, 22RV1, and PC3 cells were maintained in RPMI-1640 medium (Gibco, 11875093) supplemented with 10% fetal bovine serum (FBS; Gibco, C0232). DU145 and HEK-293T cells were cultured in Dulbecco’s Modified Eagle Medium (DMEM; Gibco, 11965092) with 10% FBS. All cultures were incubated at 37°C in a humidified atmosphere with 5% CO_2_. The small-molecule inhibitor DS18561882 was purchased from MedChemExpress (MCE, Monmouth Junction, NJ, USA) and was used in subsequent cell experiments at a final concentration of 50 μM. The phosphatase PP2A was purchased from MyBioSource (MBS1042952, San Diego, CA, USA) and was used in subsequent cell experiments at a final concentration of 10 μg/mL [22].

### 2.7 Sirna, Plasmid, and Lentivirus Transfection

siRNAs targeting UHMK1, MTHFD2, ATF4, NCOA3, and a negative control were synthesized by RiboBio (Guangzhou, China). Plasmids for UHMK1 overexpression and controls were produced by GeneChem (Shanghai, China). Transfections were conducted using Lipofectamine 2000 Reagent (Invitrogen, 11668-019, Carlsbad, CA, USA) as per the manufacturer’s instructions, with knockdown (in PC3 and DU145)/overexpression (in 22RV1) efficiency assessed by Western blotting 48 h post-transfection. Target sequences for siRNAs are provided in Table S3. For stable knockdown, lentiviruses carrying shRNA targeting UHMK1 (GV344hU6-MCS-Ubiquitin-firefly_Luciferase-IRES-puromycin) and a control vector were constructed by GeneChem. Following transfection into PC3 cells and selection with puromycin (2 μg/mL) for two weeks, stable lines were confirmed by Western blotting, using the target sequence 5^′^-GCCTATCACCTAAGAGACCTT-3^′^.

### 2.8 Cell Viability Assay

Cell viability was evaluated using Cell Counting Kit-8 (CCK8; CK04, Dojindo, Kumamoto, Japan). PC3 (5 × 10^2^ cells/well), DU145 (2 × 10^3^ cells/well) and 22RV1 (1 × 10^3^ cells/well) cells were seeded in 96-well plates and cultured for 1–5 days. At specified intervals, the medium was replaced with fresh culture medium containing 10 μL of CCK8 solution, followed by incubation for one hour at 37°C. Absorbance at 450 nm was measured with a microplate reader (Tecan, SPARK^®^10M, Männedorf, Switzerland).

### 2.9 Wound Healing Assay

Cells were seeded in 6-well plates (PC3, 1 × 10^5^ cells/well; DU145 and 22RV1, 2 × 10^5^ cells/well) and grown until 100% confluence, after which a scratch was created in the monolayer using a pipette tip. The medium was replaced with serum-free medium, and images were captured immediately and at 24 h (PC3) or 48 h (DU145 and 22RV1) post-scratch to assess wound closure, using an Olympus IX71 inverted microscope (Tokyo, Japan).

### 2.10 Cell Invasion Assay

Cell invasion was assessed using 24-well BioCoat Matrigel Invasion Chambers (8 µm pore size, Corning, NY, USA). PC3 (3 × 10^4^ cells/well), DU145 (8 × 10^4^ cells/well) and 22RV1 (5 × 10^4^ cells/well) cells were seeded in the top chamber with serum-free medium, while complete medium was added to the bottom chamber. After 24 h, invaded cells on the lower membrane surface were fixed, stained with 0.1% crystal violet, and counted under an Olympus IX71 inverted microscope (Tokyo, Japan).

### 2.11 Orthotopic Prostate Tumor Formation Assay

Four to six-week-old BALB/c nude male mice were obtained from the Laboratory Animal Center of Sun Yat-sen University. Mice were housed in a specific pathogen-free environment (24 ± 2°C, 50 ± 5% humidity, 12-h light/dark cycle) with ad libitum access to food and water. Mice were randomly divided into two groups (six per group) by using a random number table. Under isoflurane (induction, 5%; maintenance 2%) inhalation anesthesia, the bilateral testes of the mice were removed, and then stable luciferase-expressing PC3 cells (UHMK1-silenced or control cells, 1 × 10^6^ cells/mouse) were injected into the bilateral anterior prostate lobes using a syringe with a 31-gauge needle. After four weeks, bioluminescent signals were induced by intraperitoneal injection of D-luciferin (150 mg/kg) and imaged using an IVIS Spectrum System (PerkinElmer, Waltham, MA, USA). Mice were then humanely euthanized by carbon dioxide asphyxiation, and tumors were harvested for further analysis. All animal procedures complied with the ARRIVE guidelines and were approved by the Institutional Animal Care and Use Committee of Sun Yat-sen University (SYSU-IACUC-2021-000777).

### 2.12 Chromatin Immunoprecipitation (ChIP) Assay

ChIP was performed in PC3 and DU145 cells using the ChIP Assay Kit (Beyotime Biotechnology, P2078, Beijing, China) as per the manufacturer’s protocol. Cross-linked chromatin was sonicated into 200- to 1000-bp fragments and immunoprecipitated with ATF4 antibody (1:200; Cell Signaling Technology, 11815, Danvers, MA, USA) or normal rabbit IgG antibody (1:200; Proteintech, B900610). Following purification, the immunoprecipitated DNA was amplified by PCR and analyzed via agarose gel electrophoresis. PCR primers for MTHFD2 included: upstream primer: 5^′^-GTCAAGGTGTTGACAGGATTGGT-3^′^ and downstream primer: 5^′^-TCAAAGTATCTCTCCCGCAAGGA-3^′^.

### 2.13 Dual-Luciferase Reporter Assay

A dual-luciferase reporter assay was conducted in HEK-293T cells. Firefly promoter–luciferase reporter constructs (0.5 µg, MTHFD2-WT or MTHFD2-mutant promoter) were co-transfected with ATF4 plasmids (0.5 µg) using Lipofectamine 2000 Reagent (11668-019, Invitrogen). Renilla luciferase reporter (0.5 µg) served as an internal control. Luciferase activity was measured using a Dual-Luciferase Reporter Assay System (E1910, Promega, Madison, WI, USA). The ATF4 plasmids, firefly promoter–luciferase reporters and renilla luciferase reporter used above were constructed by GeneChem.

### 2.14 Coimmunoprecipitation (CO-IP) Assay

Briefly, PC3 or DU145 cells were lysed using a Whole Cell Lysis Assay (KeyGenBioTECH, KGP250) and the lysates were incubated with NCOA3 antibody (1:50; Abcam, ab2831, Cambridge, MA, USA) or normal rabbit IgG antibody (1:50; Proteintech, B900610) overnight at 4°C. Then the complexes were immunoprecipitated using 20 µL Protein A+G Agarose (P2055, Beyotime Biotechnology) for three hours at 4°C. Following washes, samples were eluted with SDS-PAGE sample loading buffer (2×, pH = 6.8; P0015A, Beyotime Biotechnology) and boiled before Western blot analysis.

### 2.15 Bioinformatic Tools

Metabolism-related gene sets were obtained from the GSEA Molecular Signatures Database (MSigDB, https://www.gsea-msigdb.org/gsea/msigdb/index.jsp) (accessed on 8 July 2025). The raw sequencing expression matrices were converted by FPKM, and then each gene was Z-score normalized in the row direction to achieve comparability of expression levels among different genes. Finally, heatmaps were plotted using the pheatmap package in R (version 4.2.2, R Foundation, Vienna, Austria) to visualize the average expression levels of metabolism-related genes after UHMK1 knockdown. For heatmap visualization, sample R1802792 represents the negative control group (si-Con), and R1802791 represents the UHMK1 knockdown group (si-UHMK1). The potential binding sites of ATF4 in the promoter region of MTHFD2 were predicted using the JASPAR database (https://jaspar.genereg.net/) (accessed on 8 July 2025).

### 2.16 Statistical Analysis

Statistical analyses were conducted using IBM SPSS Statistics 23.0 (IBM Corporation, Armonk, NY, USA). All *in vitro* experiments were performed in triplicate, with all data presented as mean ± standard deviation (SD). Differences between two groups were assessed using Student’s *t*-test, while one-way ANOVA followed by a Student–Newman–Keuls post hoc test was employed for multiple comparisons. The association between UHMK1 expression and clinicopathological characteristics was analyzed using the Chi-square test. Survival analysis was performed using the log-rank test based on Kaplan–Meier methods. A two-tailed *p*-value < 0.05 was considered statistically significant.

## 3 Results

### 3.1 Elevated UHMK1 Expression in PCa Positively Correlates with Tumor Malignancy

**Figure 1:**
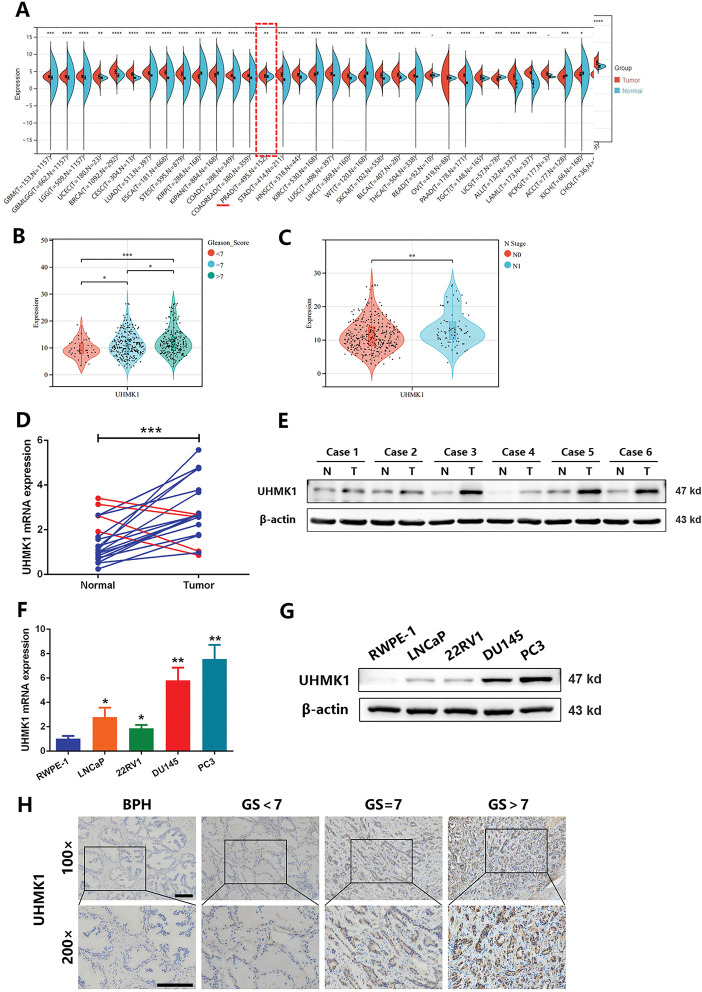

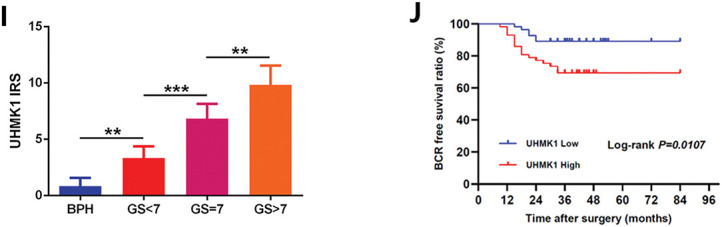
U2AF Homology Motif Kinase 1 (UHMK1) is highly expressed in prostate cancer (PCa) and positively correlates with tumor malignancy. **(A)** UHMK1 mRNA expression levels across various cancers from the TCGA and GTEx datasets. **(B)** UHMK1 mRNA expression levels in PCa tissues with different Gleason scores from the TCGA database. **(C)** UHMK1 mRNA expression levels in stage N0-N1 PCa tissues from the TCGA database. **(D)** RT-qPCR analysis of UHMK1 mRNA expression in 20 pairs of PCa tumor tissues and adjacent normal tissues. Blue lines indicate an increased UHMK1 expression in tumors, whereas red lines indicate a decreased UHMK1 expression in tumors. **(E)** Western blot analysis of UHMK1 protein expression in pairs of matched PCa (T) and adjacent normal tissues (N). **(F)** RT-qPCR analysis of UHMK1 mRNA expression in different PCa cell lines. **(G)** Western blot analysis of UHMK1 protein expression in different PCa cell lines. **(H, I)** Representative IHC staining of UHMK1 in BPH and PCa tissues with different Gleason scores (GS) (scale bar = 100 μm; n = 6 per group). **(J)** Kaplan-Meier analysis of BCR-free survival for PCa patients with differential expression of UHMK1. *p < 0.05; **p < 0.01; ***p < 0.001; ****p < 0.0001

Based on both TCGA and GTex public datasets, UHMK1 expression levels were significantly higher in PCa samples compared to normal samples. Increased UHMK1 expression was observed in patients with high Gleason scores and lymph node metastasis (Fig. 1A–C). RT-qPCR and Western blotting analyses of UHMK1 expression in PCa tissues and adjacent normal tissues showed that both mRNA and protein levels of UHMK1 were significantly elevated in PCa tissues compared to paracancerous tissues (Fig. 1D,E). *In vitro*, evaluations across PCa cell lines (LNCaP, 22RV1, DU145, PC3) compared to normal prostatic epithelial cells (RWPE-1) also displayed significantly heightened UHMK1 expression at both transcript and protein levels (Fig. 1F,G). IHC further validated UHMK1 upregulation in PCa compared to BPH tissues. Crucially, UHMK1 expression was strongly associated with Gleason score, tumor stage, and lymph node metastasis status, but not patient age (Figs. 1H,I and Table S4). Moreover, Kaplan–Meier analysis of our single-center cohort study involving 112 patients indicated that PCa patients with higher levels of UHMK1 had poorer biochemical recurrence (BCR)-free survival (Fig. 1J). Collectively, these findings establish UHMK1 overexpression in PCa as a feature linked to tumor malignancy.

### 3.2 UHMK1 Promotes PCa Cell Proliferation and Invasion In Vitro and In Vivo

**Figure 2:**
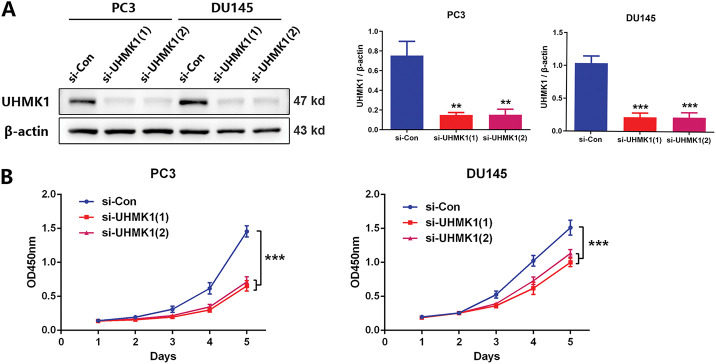

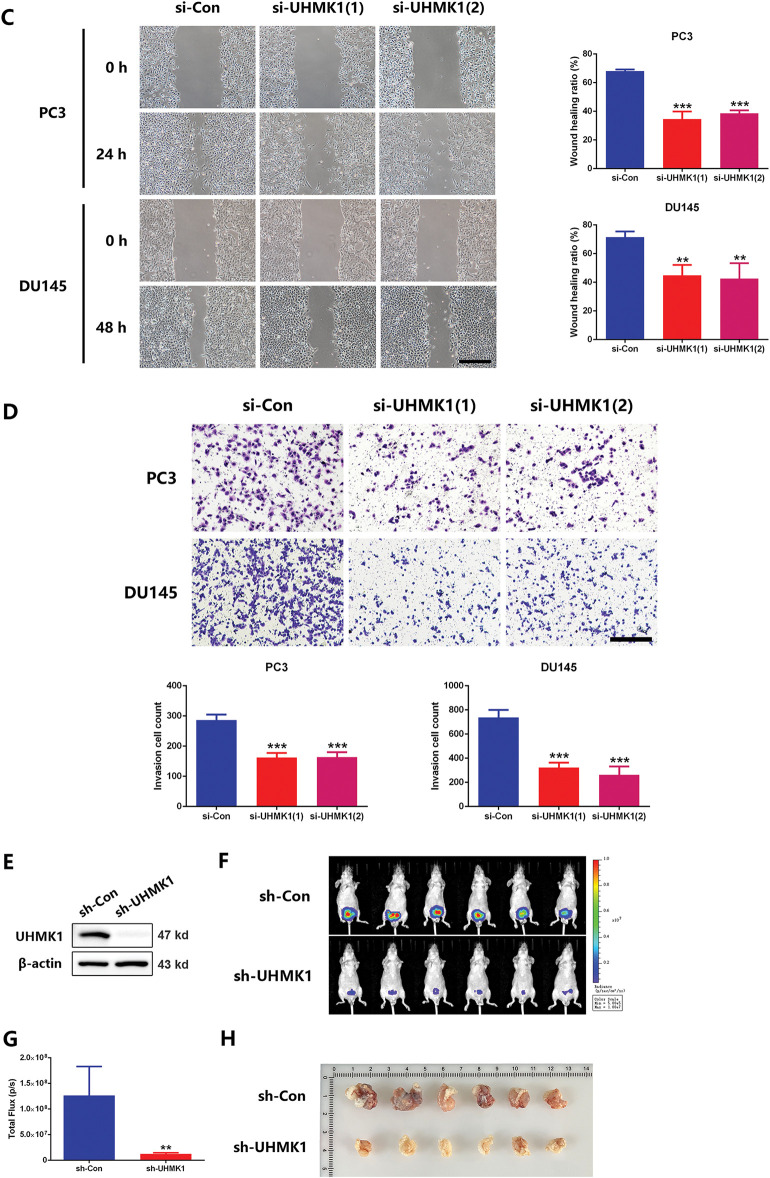
UHMK1 promotes PCa cell proliferation and invasion *in vitro* and *in vivo*. **(A)** Western blot analysis demonstrating the efficiency of UHMK1 knockdown in PC3 and DU145 cells. **(B)** CCK8 assay to assess the proliferation ability of PC3 and DU145 cells following UHMK1 knockdown. **(C)** Wound healing assay to assess the migratory capacity of PC3 and DU145 cells following UHMK1 knockdown (scale bar = 400 μm). **(D)** Cell invasion assay to assess the invasive capacity of PC3 and DU145 cells following UHMK1 knockdown (scale bar = 200 μm). **(E)** Western blot analysis demonstrating the efficiency of UHMK1 silencing in PC3 cells. **(F, G)** Bioluminescent images of tumor formation following xenografting of PC3 cells with UHMK1 silencing (n = 6 per group). **(H)** Gross images of tumors extracted from xenograft mice. **p < 0.01; ***p < 0.001

To delineate UHMK1’s functional role in PCa, we knocked down UHMK1 expression in PC3 and DU145 cells and overexpressed UHMK1 in 22RV1 cells (Fig. 2A; Fig. S1A). Western blot analysis showed that the knockdown efficiency of the two siRNA copies against UHMK1 was 80.2% and 79.8% in PC3 cells and 79.0% and 79.0% in DU145 cells, respectively. Depletion of UHMK1 significantly impaired the proliferation of PC3 and DU145 cells, whereas its overexpression enhanced proliferation in 22RV1 cells (Fig. 2B; Fig. S1B). Similarly, wound healing and cell invasion assays demonstrated that the migration and invasion abilities were decreased in PC3 and DU145 cells after UHMK1 knockdown but increased in 22RV1 cells after UHMK1 overexpression (Fig. 2C,D; Fig. S1C,D). To further evaluate the effect of UHMK1 on PCa tumorigenicity *in vivo*, we established an orthotopic human prostate tumor model by injecting stably silenced UHMK1 and control PC3 cells. UHMK1-silenced PC3 cells exhibited reduced tumorigenicity and formed smaller tumors compared to the control cells (Figs. 2E–H). Taken together, these data suggest that UHMK1 promotes PCa progression *in vitro* and *vivo*.

### 3.3 MTHFD2 Mediates the Role of UHMK1 in Promoting PCa Progression

**Figure 3:**
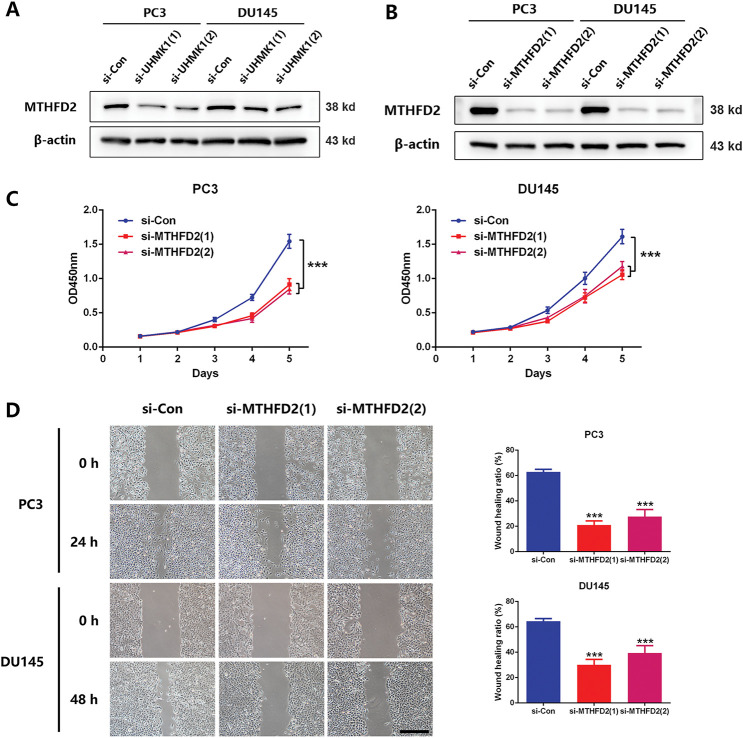

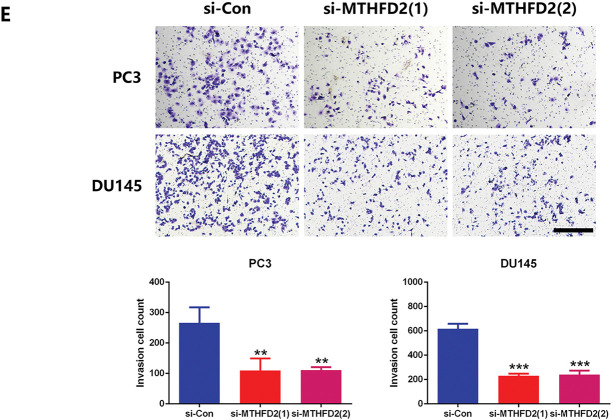
Methylenetetrahydrofolate Dehydrogenase (MTHFD2) mediates the role of UHMK1 in promoting PCa progression. **(A)** Western blot analysis of the protein levels of MTHFD2 following UHMK1 knockdown in PC3 and DU145 cells. **(B)** The confirmation of MTHFD2 knockdown in PC3 and DU145 cells by Western blot. **(C)** CCK8 assay to measure the proliferation ability of PC3 and DU145 cells following MTHFD2 knockdown. **(D)** Wound healing assay to measure the migratory capacity of PC3 and DU145 cells following MTHFD2 knockdown (scale bar = 400 μm). **(E)** Cell invasion assay to determine the invasive potential of PC3 and DU145 cells following MTHFD2 knockdown (scale bar = 200 μm). **p < 0.01; ***p < 0.001

Based on NGS data, we identified a panel of nucleotide metabolism-related genes that may be regulated by UHMK1 (Fig. S2A). Subsequent RT-qPCR validation in UHMK1-knockdown PC3 and DU145 cells confirmed downregulation of PHGDH, PSAT1, PSPH, MTHFD2, DHFR, PPAT, and TYMS (Fig. S2B). Western blot assays showed that MTHFD2 expression was reduced after UHMK1 knockdown in PC3 and DU145 cells (Fig. 3A,B). Further *in vitro* experiments revealed that MTHFD2 knockdown inhibited the proliferation, migration and invasion of PC3 and DU145 cells (Fig. 3C–E). Moreover, DS18561882, a highly potent isozyme-selective inhibitor of MTHFD2, was capable of abrogating the aggressive behavior induced by UHMK1 overexpression in 22RV1 cells (Fig. S2C-F). All of the above results suggest that MTHFD2 is a downstream regulatory target of UHMK1 and mediates the role of UHMK1 in promoting PCa progression.

### 3.4 UHMK1 Mediates MTHFD2 Expression via Activating ATF4

**Figure 4:**
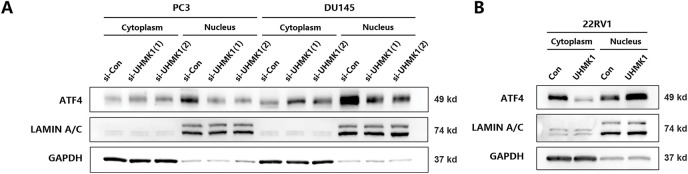

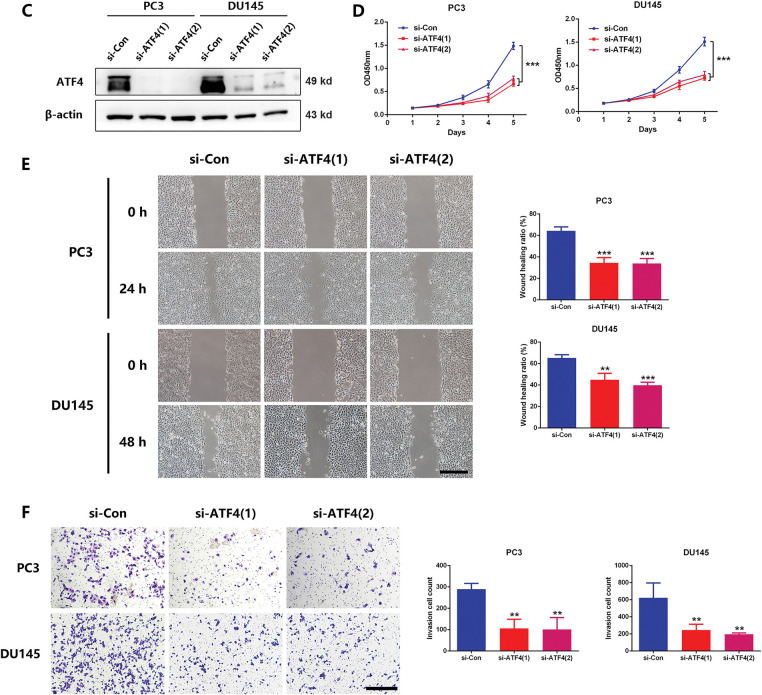
UHMK1 promotes the activation of Activating Transcription Factor 4(ATF4) to initiate MTHFD2 expression. **(A)** Western blot analysis of cytoplasmic and nuclear protein levels of ATF4 following UHMK1 knockdown in PC3 and DU145 cells. **(B)** Western blot analysis of cytoplasmic and nuclear protein levels of ATF4 following UHMK1 overexpression in 22RV1 cells. **(C)** Confirmation of ATF4 knockdown in PC3 and DU145 cells by Western blotting. **(D)** CCK8 assay to assess the proliferation ability of PC3 and DU145 cells following ATF4 knockdown. **(E)** Wound healing assay to assess the migratory ability of PC3 and DU145 cells following ATF4 knockdown (scale bar = 40 μm). **(F)** Cell invasion assay to assess the invasive ability of PC3 and DU145 cells following ATF4 knockdown (scale bar =200 μm). **p < 0.01; ***p < 0.001

It has been reported that the expression of MTHFD2 was dependent on the stimulation of ATF4, a transcription factor whose nuclear translocation is essential for its transcriptional activity [23]. Therefore, we speculate whether ATF4 is involved in the regulation of MTHFD2 by UHMK1. Western blot analyses revealed that the nuclear protein level of ATF4 was reduced after UHMK1 knockdown in PC3 and DU145 cells, whereas the nuclear protein level of ATF4 was increased after UHMK1 overexpression in 22RV1 cells, indicating that UHMK1 influences the nuclear translocation of ATF4 (Fig. 4A,B). Additionally, knockdown of ATF4 significantly decreased the proliferation, migration, and invasion of PCa cells (Fig. 4C–F).

To investigate whether ATF4 can transcriptionally activate MTHFD2 in PC3 cells, we first performed RT–qPCR and Western blot assays, which revealed that MTHFD2 mRNA and protein levels were decreased following ATF4 knockdown in PC3 and DU145 cells (Fig. S3A,B). Using the JASPAR database, then, we identified two potential ATF4 binding sites in the MTHFD2 promoter region (Fig. S3C). Mutation of these sites led to a significant reduction in MTHFD2-luciferase reporter activity, suggesting that ATF4 can transcriptionally activate the MTHFD2 promoter through these binding sites (Fig. S3D). Furthermore, ChIP assays confirmed that the ATF4 directly interacts with the MTHFD2 promoter (Fig. S3E). These findings indicate that UHMK1 enhances ATF4 activation, thereby initiating MTHFD2 expression in prostate cancer cells.

### 3.5 UHMK1-Induced Phosphorylation of NCOA3 Is Responsible for the Activation of ATF4

**Figure 5:**
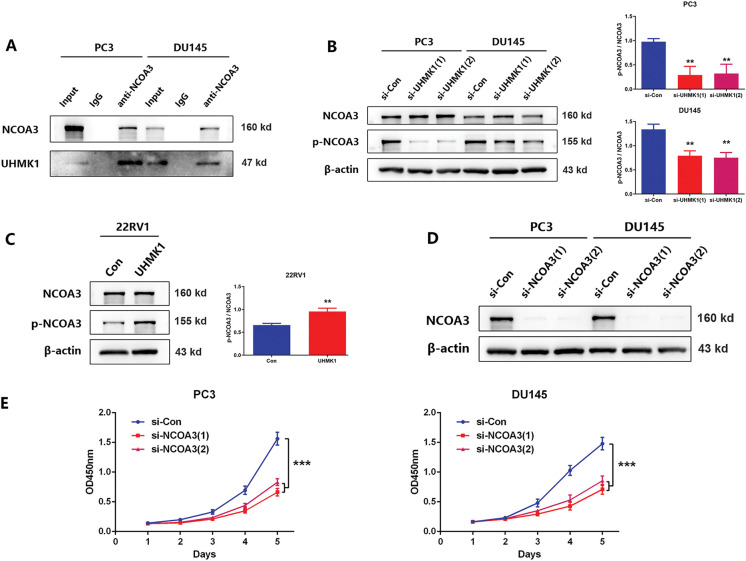

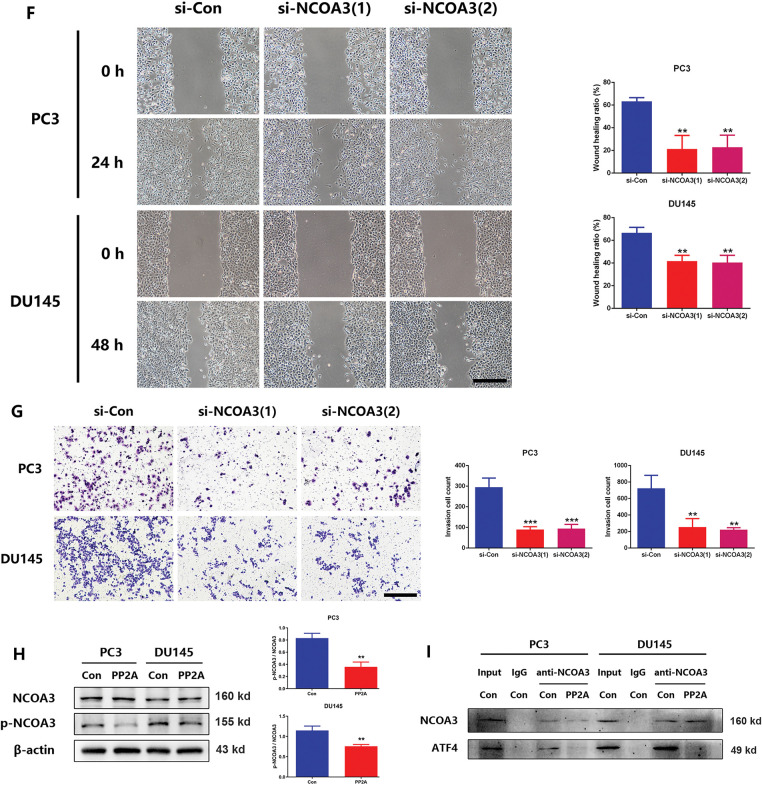

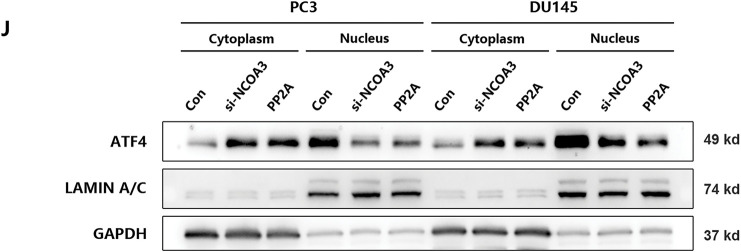
UHMK1-induced phosphorylation of Nuclear Receptor Coactivator 3(NCOA3) was responsible for the activation of ATF4. **(A)** A Coimmunoprecipitation (CO-IP) assay revealed an interaction between endogenously expressed NCOA3 and UHMK1 in PC3 and DU145 cells. **(B, C)** Western blotting analyses were performed to evaluate NCOA3 and serine 857-phosphorylated NCOA3 protein expression following UHMK1 knockdown in PC3 and DU145 cells, and UHMK1 overexpression in 22RV1 cells. **(D)** Confirmation of NCOA3 knockdown in PC3 and DU145 cells by Western blot. **(E)** CCK8 assay to assess the proliferation ability of PC3 and DU145 cells following NCOA3 knockdown. **(F)** Wound healing assay to assess the migratory ability of PC3 and DU145 cells following NCOA3 knockdown (scale bar = 400 μm). **(G)** Cell invasion assay to evaluate the invasive ability of PC3 and DU145 cells following NCOA3 knockdown (scale bar = 200 μm). **(H)** Western blotting analyses were performed to evaluate NCOA3 and phosphorylated NCOA3 protein expression following PP2A treatment in PC3 and DU145 cells. **(I)** CO-IP assay to evaluate the binding of NCOA3 to ATF4 and the effect of PP2A on this binding. **(J)** Western blotting analyses were performed to assess cytoplasmic and nuclear ATF4 protein expression following NCOA3 knockdown or PP2A treatment in PC3 and DU145 cells. **p < 0.01; ***p < 0.001

To explore the mechanism by which UHMK1 promotes the nuclear translocation of ATF4, we focused on NCOA3, a well-established coactivator of ATF4. CO-IP assays revealed an interaction between endogenous NCOA3 and UHMK1 in PCa cells (Fig. 5A). Western blot results demonstrated that phosphorylation levels of NCOA3 at serine 857 decreased following UHMK1 knockdown in PC3 and DU145 cells, whereas these levels increased following UHMK1 overexpression in 22RV1 cells, indicating that UHMK1 regulates NCOA3 phosphorylation at serine 857 (Fig. 5B,C). Furthermore, NCOA3 knockdown in PCa cells resulted in inhibition of cell proliferation, migration and invasion (Figs. 5D–G). These findings suggest that NCOA3 may be involved in UHMK1-mediated cancer progression.

Recent studies have shown that phosphorylated NCOA3 is recruited by ATF4 to form transcriptional complexes, initiating nuclear translocation and regulating downstream gene expression [24]. To verify that phosphorylated NCOA3 binds to ATF4, PP2A (a phosphatase that inhibits NCOA3 phosphorylation) was used to pretreat PCa cells [25]. Following PP2A treatment, NCOA3 phosphorylation at serine 857 was significantly reduced in PC3 and DU145 cells (Fig. 5H). CO-IP assays revealed that NCOA3 directly binds to ATF4, and this interaction was inhibited by PP2A (Fig. 5I). Subsequently, we focused on whether phosphorylated NCOA3 induces nuclear translocation of ATF4 and used PP2A to inhibit this process *in vitro*. Western blot analyses further confirmed that the nuclear translocation of ATF4 was significantly blocked following PP2A treatment, which inhibited NCOA3 phosphorylation (Fig. 5J). These results suggested that UHMK1-dependent phosphorylation of NCOA3 at serine 857 may be responsible for initiating ATF4 activation.

### 3.6 A Positive Feedback Loop Exists between UHMK1 and MTHFD2

**Figure 6:**
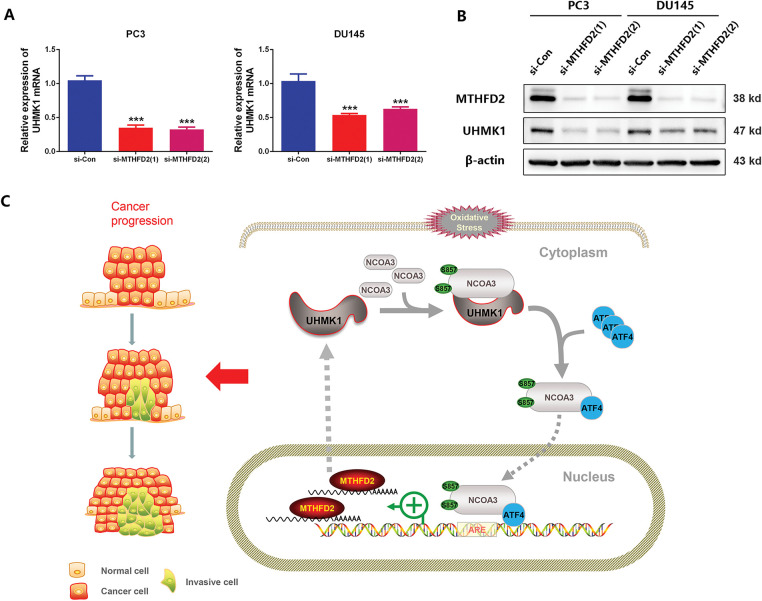
A positive feedback loop exists between UHMK1 and MTHFD2. **(A)** RT-qPCR was performed to assess the mRNA level of UHMK1 following MTHFD2 knockdown in PC3 and DU145 cells. **(B)** Western blotting was performed to evaluate UHMK1 protein expression under the same conditions. **(C)** A schematic illustrating the role of the UHMK1/MTHFD2 positive feedback loop in PCa progression (created using ScienceSlides-PowerPoint (PPT) software). ***p < 0.001

Previously, we demonstrated that UHMK1 enhances MTHFD2 expression by promoting NCOA3 phosphorylation and ATF4 nuclear translocation. Interestingly, we also observed that silencing MTHFD2 in PC3 and DU145 cells led to significant reductions in both the mRNA and protein levels of UHMK1 (Fig. 6A,B). These findings suggest that MTHFD2 regulates UHMK1 expression. Collectively, our results indicate the existence of a positive feedback loop between UHMK1 and MTHFD2, sustaining continuous activation of this signaling pathway and facilitating tumor progression (Fig. 6C).

## 4 Discussion

UHMK1 dysregulation or mutation represents a high-penetrance factor implicated in diverse cancers [26–28]. In colorectal cancer, hyperactivating UHMK1/STAT3 positive feedback loop contributes to colorectal cancer cell proliferation and chemoresistance. Additionally, EBLN3P overexpression enhances tumorigenesis by attenuating miR-323a-3p-mediated suppression of UHMK1 [29,30]. Studies investigating the role of Ser/Thr kinase in the progression of PCa have gained increasing attention in recent years [31–33]. However, the biological function of UHMK1, a Ser/Thr kinase, in the development of PCa remains unclear. In our study, we observed that UHMK1 was aberrantly overexpressed in PCa, a finding further confirmed by public TCGA and GTEx datasets. Analysis of our 7-year follow-up data revealed that higher UHMK1 expression was significantly correlated with poorer BCR-free survival. Furthermore, we demonstrated that UHMK1 upregulation in PCa significantly enhanced tumor growth and dissemination. Collectively, these findings suggest that UHMK1 functions as an oncogene and is associated with poor prognosis.

As mentioned earlier, UHMK1 has been recognized as a critical downstream effector of many signaling pathways that drive cancer proliferation and invasion [34–36]. We suggest that the unidentified downstream signaling cascade of UHMK1 is essential for regulating the aforementioned processes. Recently, Feng et al. found that UHMK1 enhances gastric cancer progression by increasing *de novo* purine synthesis, indicating metabolic reprogrammed as a key factor in cancer progression [37]. In this study, we identified several potential downstream targets of UHMK1 in PCa related to serine, purine, and amino acid metabolism and one-carbon metabolism through transcriptome sequencing. These pathways are essential for nucleotide synthesis, suggesting that UHMK1 may also be involved in nucleotide metabolic reprogramming in PCa. Given that serine, purine and amino acid metabolism supply precursors for one-carbon metabolism, we hypothesize that targeting one-carbon metabolism could effectively suppress tumor growth [38]. Therefore, we focused on MTHFD2, a key regulator in the mitochondrial one-carbon pathway, which is overexpressed in various tumors [39–41]. In lung cancer, MTHFD2 is activated by ATF4 to maintain redox homeostasis and contributes to cancer progression by regulating AKT/GSK-3β/β-catenin signaling [42,43]. In PCa, we found that MTHFD2 knockdown significantly inhibited cell aggression, likely due to its roles in nucleotide synthesis, S-adenosyl methionine biosynthesis and redox homeostasis. Moreover, MTHFD2 inhibition reversed UHMK1-induced PCa aggressiveness, suggesting that MTHFD2 is critical for UHMK1-mediated behaviors. Interestingly, we also discovered that MTHFD2 could regulate UHMK1 expression. Possibly through specific transcription factors, warranting further investigation. This study is the first to propose a UHMK1/MTHFD2 positive feedback loop in PCa progression, potentially involving nucleotide metabolism reprogramming.

Mechanistically, we identified ATF4 as a downstream effector of UHMK1. Tameire et al. showed that c-myc drives ATF4 nuclear enrichment to meet bioenergetic demands during tumor progression [44]. In this study, UHMK1 knockdown enhanced ATF4 nuclear translocation without affecting its mRNA levels, suggesting that UHMK1 is crucial for ATF4 activation. Moreover, Ben-Sahra et al. revealed that ATF4 stimulates MTHFD2 to enhance *de novo* purine synthesis in several cancers [45]. Here, we found that ATF4 knockdown inhibited MTHFD2 expression and restrained PCa cell growth and dissemination. Promoter sequence analysis revealed two potential ATF4 binding sites in the MTHFD2 promoter, and dual-luciferase reporter and ChIP assays confirmed direct interaction between ATF4 and the MTHFD2 promoter. These data strongly suggest that ATF4 is a key signal transducer in the UHMK1/MTHFD2 positive feedback loop.

NCOA3 is a coactivator that collaborates with transcription factors in tumorigenesis [46]. Gupta et al. suggested that NCOA3 is the key effector in activating the PERK-ATF4 pathway in breast cancer [24]. Our data concluded that NCOA3 binds to ATF4, forming a protein complex that acts as a direct coactivator for ATF4 activation. Given that hyperactivation of NCOA3 is common in many tumors, its posttranslational modification, particularly phosphorylation, has recently gained increasing attention [47–49]. It has been noted that phosphorylation of NCOA3 is required for the regulation of its transcriptional activity, which is closely correlated with protein stability, flexibility and subcellular localization [50,51]. In a previous report, phosphorylated NCOA3 at serine 857 has been shown to enhance ATF4 transcriptional activity [23,52]. Our findings demonstrate that phosphorylation of NCOA3 at serine 857 plays a role in UHMK1-mediated ATF4 transcriptional activity in PCa. However, additional experimental studies are required to confirm whether phosphorylation at this specific site is essential for ATF4 activation.

This study has certain limitations. We identified a positive feedback loop between UHMK1 and MTHFD2 and confirmed that UHMK1 enhances MTHFD2 expression by promoting NCOA3 phosphorylation and ATF4 nuclear translocation. However, the specific mechanism by which MTHFD2 reciprocally regulates UHMK1 expression remains unclear, particularly given that MTHFD2 is primarily localized to the mitochondria, whereas UHMK1 is in the nucleus and cytoplasm. Additionally, the exact phosphorylation site of NCOA3 induced by UHMK1 requires further investigation. Furthermore, although we explored the role of UHMK1, an oncoprotein, in PCa as a potential therapeutic target, the literature on specific chemical inhibitors targeting UHMK1 is limited. Based on current research, certain known compounds may offer valuable insights for future research. For instance, Le et al. reported that trastuzumab can concentration-dependently inhibit UHMK1 expression in breast cancer [53]. Additionally, FMF-06-098-1, a multitargeted inhibitor, promotes the degradation of UHMK1 via a kinase degradation pathway [54]. All these compounds’ clinical efficacy requires further validation. These issues represent critical directions for our future research.

## 5 Conclusions

This study provides strong evidence that UHMK1 contributes to PCa progression through a UHMK1/MTHFD2 positive feedback loop, suggesting that UHMK1 might be a potential biomarker and attractive therapeutic target for advanced PCa.

## Supplementary Materials

Figure S1 **UHMK1 promotes PCa cell proliferation and invasion in vitro and in vivo.** **(A)** Western blot analysis of UHMK1 overexpression efficiency in 22RV1 cells. **(B)** CCK8 assay to measure the proliferation ability of 22RV1 cells after UHMK1 overexpression. **(C)** Wound healing assay to measure the migration ability of 22RV1 cells after UHMK1 overexpression (scale bar =400 μm). **(D)** Cell invasion assay to measure the invasion ability of 22RV1 cells after UHMK1 overexpression (scale bar =200 μm). **p < 0.01; *** *p*< 0.001.

Figure S2 **MTHFD2 mediates the role of UHMK1 in promoting PCa progression.** **(A)** RNA-seq was used to examine nucleotide metabolism-related genes in PC3 cells with or without UHMK1 knockdown. The data are shown in the heatmap (R1802792: si-Con; R1802791: si-UHMK1). **(B)** RT-qPCR was used to measure the mRNA levels of UHMK1, PHGDH, PSAT1, PSPH, MTHFD2, DHFR, PPAT and TYMS after depletion of UHMK1 in PC3 and DU145 cells. **(C)** Western blot analysis of expression of MTHFD2 after UHMK1 overexpression in 22RV1 cells. **(D)** CCK8 assay to measure the proliferation ability of 22RV1 cells overexpressing UHMK1 after treatment with an MTHFD2 inhibitor (DS18561882, 50 µM). **(E)** Wound healing assay to measure the migration ability of 22RV1 cells overexpressing UHMK1 after treatment with an MTHFD2 inhibitor (scale bar =400 μm). **(F)** Cell invasion assay to measure the invasion ability of 22RV1 cells overexpressing UHMK1 after treatment with an MTHFD2 inhibitor (scale bar =400 μm). * *p* < 0.05; ** *p* < 0.01; *** *p*< 0.001.

Figure S3 **ATF4 interacts with the MTHFD2 promoter to activate transcription.** **(A)** RT-qPCR was used to measure the mRNA level of MTHFD2 after ATF4 knockdown in PC3 and DU145 cells. **(B)** Western blot analysis of MTHFD2 protein expression after ATF4 knockdown in PC3 and DU145 cells. **(C)** ATF4 motif consensus sequence from JASPAR. The nucleotide sequences of the -1,494/-897 region in the human MTHFD2 gene and the candidate ATF4 binding sites (red text). **(D)** Dual-luciferase reporter assay to evaluate the effect of ATF4 on the activity of the WT-MTHFD2 promoter and its mutants. **(E)** ChIP assay to verify the binding of ATF4 to the MTHFD2 promoter in PC3 and DU145 cells. *p < 0.05; ** *p* < 0.01; *** *p* < 0.001.

## Data Availability

The datasets used and/or analyzed during the current study are available from the corresponding authors on reasonable request.

## Supplementary Materials

The supplementary material is available online at https://www.techscience.com/doi/10.32604/or.2025.065119/s1.

## Abbreviations

UHMK1: U2AF Homology Motif Kinase 1
NCOA3: Nuclear Receptor Coactivator 3
MTHFD2: Methylenetetrahydrofolate Dehydrogenase 2
SF1: Splicing Factor 1
AMPA: α-Amino-3-Hydroxy-5-Methyl-4-Isoxazolepropionic Acid
ATF4: Activating Transcription Factor 4
PCa: Prostate Cancer
IHC: Immunohistochemistry
ADT: Androgen Deprivation Therapy
CRPC: Castration-Resistant Prostate Cancer
NGS: Next-Generation Sequencing
BPH: Benign Prostatic Hyperplasia
IRS: Immunoreactive Score
ECL: Enhanced Chemiluminescence
ATCC: American Type Culture Collection
FBS: Fetal Bovine Serum
SPF: Specific Pathogen-Free
Chip: Chromatin Immunoprecipitation
CO-IP: Coimmunoprecipitation
SD: Standard Deviation
BCR: Biochemical Recurrence
