# Supplementary figures and images for: UHMK1 Promotes Prostate Cancer Progression through a Positive Feedback Loop with MTHFD2
Source: Oncol Res. 2025 Aug 28;33(9):2331–51. doi: 10.32604/or.2025.065119 (PMC12408860; doi:10.32604/or.2025.065119)

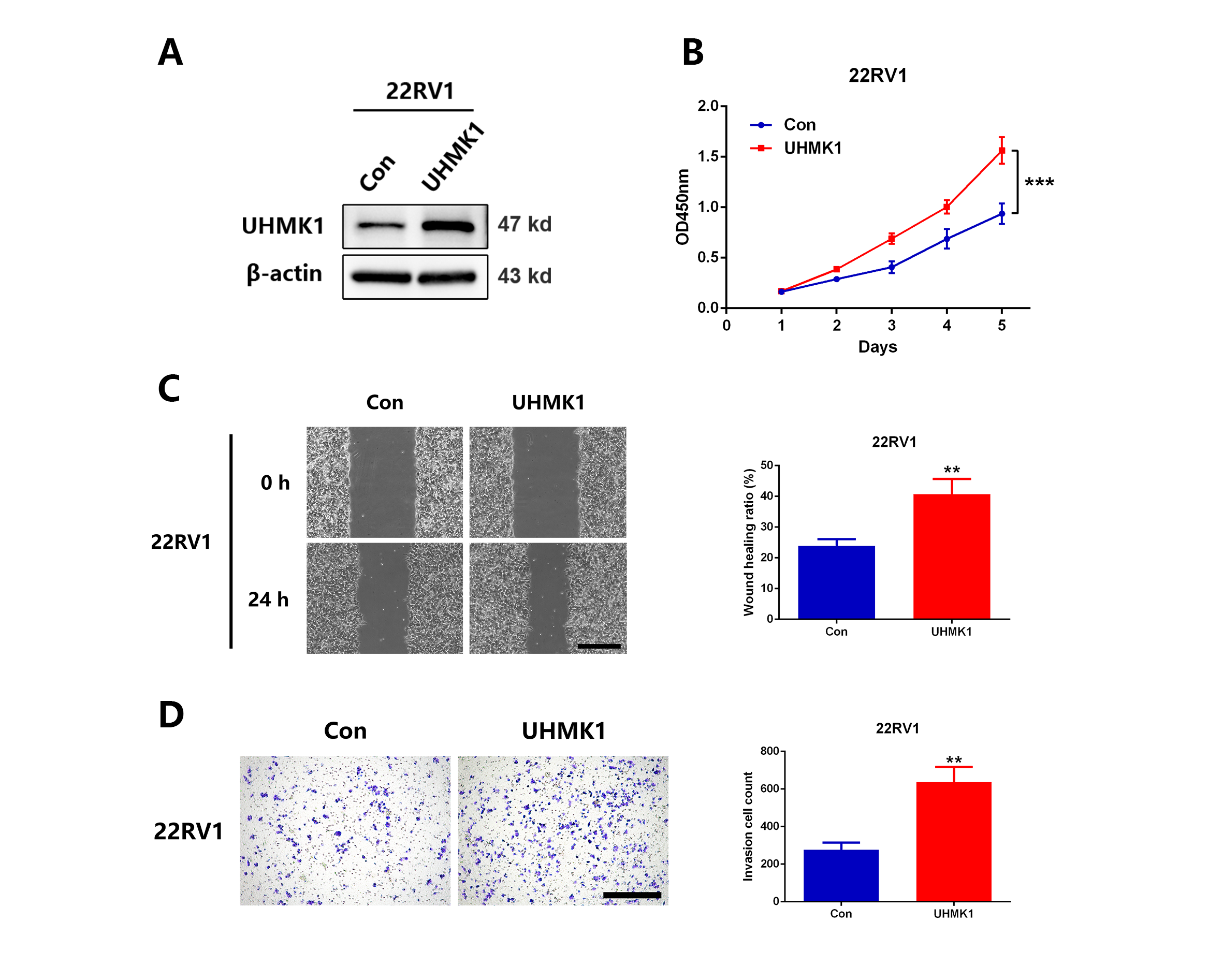

Supplement: Figure S1 [file OncolRes-33-65119-s001.tif]

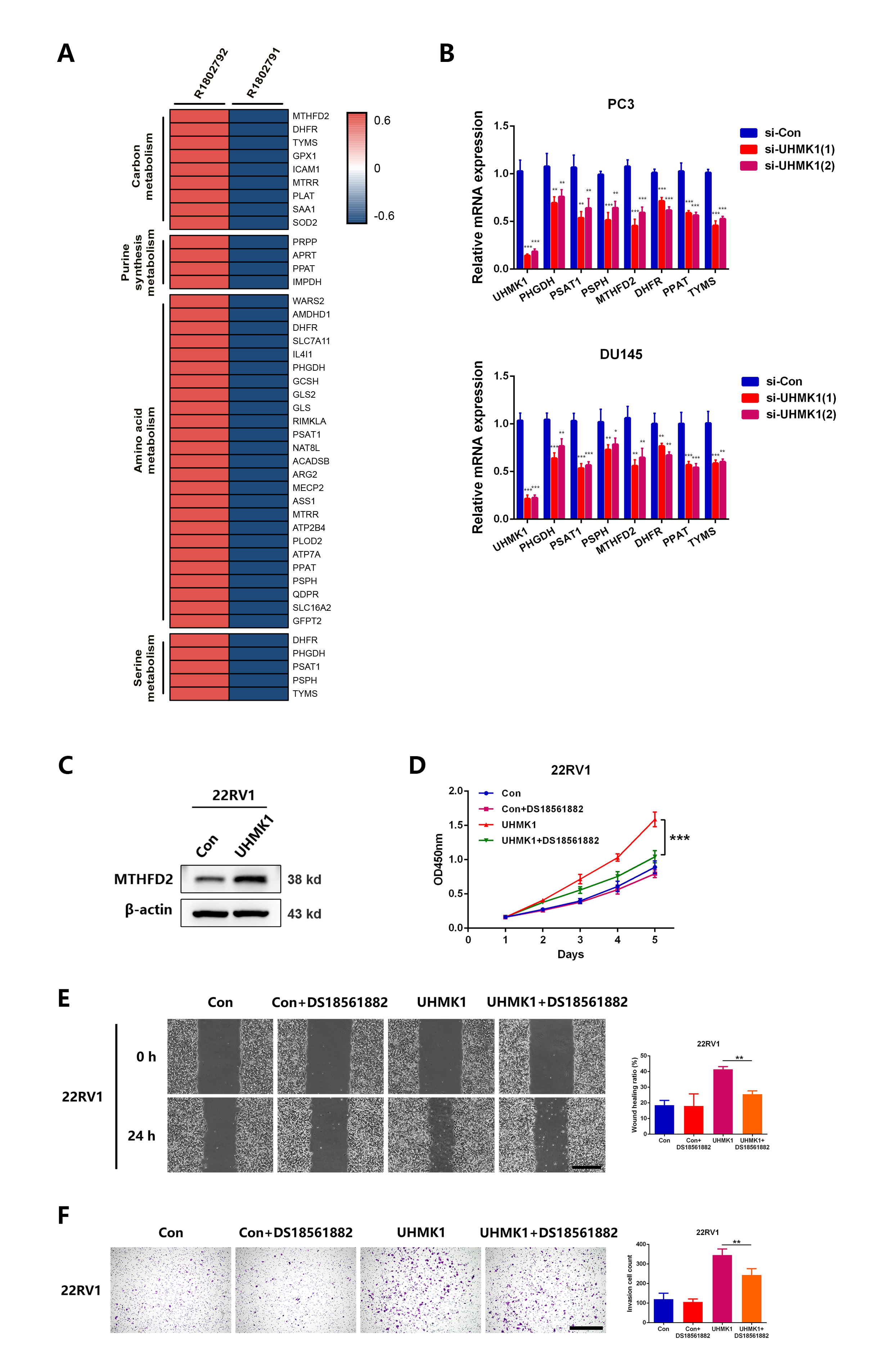

Supplement: Figure S2 [file OncolRes-33-65119-s002.tif]

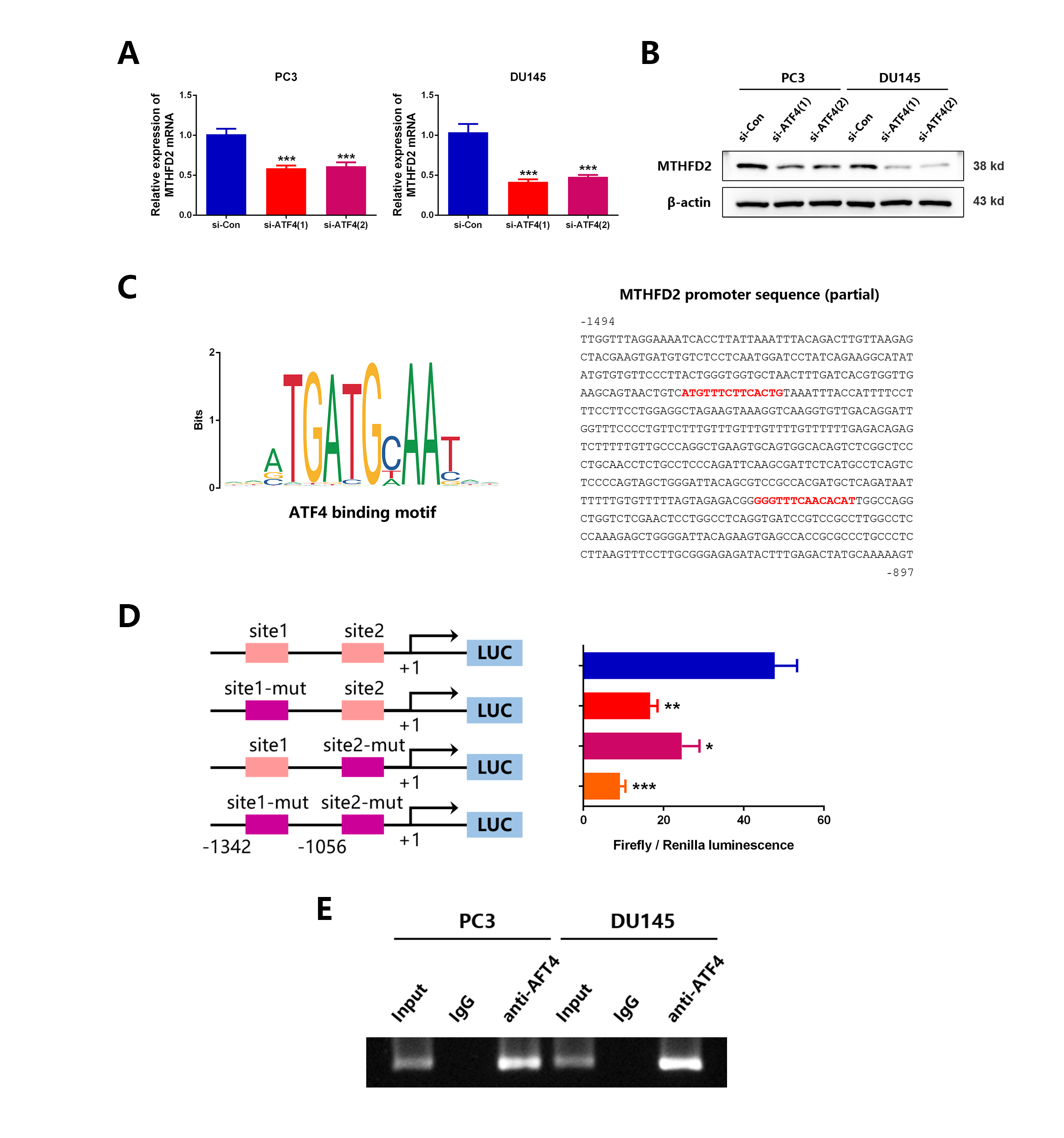

Supplement: Figure S3 [file OncolRes-33-65119-s003.tif]
