# Supplementary material for: UHMK1 Promotes Prostate Cancer Progression through a Positive Feedback Loop with MTHFD2
Source: Oncol Res. 2025 Aug 28;33(9):2331–51. doi: 10.32604/or.2025.065119 (PMC12408860; doi:10.32604/or.2025.065119)
Supplement: Supplementary file 4 [file OncolRes-33-65119-s004.docx]

**Figure S1. UHMK1 promotes PCa cell proliferation and invasion *in vitro* and in *vivo*.**

**(A)** Western blot analysis of UHMK1 overexpression efficiency in 22RV1 cells. **(B)** CCK8 assay to measure the proliferation ability of 22RV1 cells after UHMK1 overexpression. **(C)** Wound healing assay to measure the migration ability of 22RV1 cells after UHMK1 overexpression (scale bar =400 μm). **(D)** Cell invasion assay to measure the invasion ability of 22RV1 cells after UHMK1 overexpression (scale bar =200 μm). ***p* < 0.01; *** *p*< 0.001.

**Figure S2. MTHFD2 mediates the role of UHMK1 in promoting PCa progression. (A)** RNA-seq was used to examine nucleotide metabolism-related genes in PC3 cells with or without UHMK1 knockdown. The data are shown in the heatmap (R1802792: si-Con; R1802791: si-UHMK1). **(B)** RT-qPCR was used to measure the mRNA levels of UHMK1, PHGDH, PSAT1, PSPH, MTHFD2, DHFR, PPAT and TYMS after depletion of UHMK1 in PC3 and DU145 cells. **(C)** Western blot analysis of expression of MTHFD2 after UHMK1 overexpression in 22RV1 cells. **(D)** CCK8 assay to measure the proliferation ability of 22RV1 cells overexpressing UHMK1 after treatment with an MTHFD2 inhibitor (DS18561882, 50 µM). **(E)** Wound healing assay to measure the migration ability of 22RV1 cells overexpressing UHMK1 after treatment with an MTHFD2 inhibitor (scale bar =400 μm). **(F)** Cell invasion assay to measure the invasion ability of 22RV1 cells overexpressing UHMK1 after treatment with an MTHFD2 inhibitor (scale bar =400 μm). * *p* < 0.05; ** *p* < 0.01; *** *p* < 0.001.

**Figure. S3 ATF4 interacts with the MTHFD2 promoter to activate transcription.**

**(A)** RT-qPCR was used to measure the mRNA level of MTHFD2 after ATF4 knockdown in PC3 and DU145 cells. **(B)** Western blot analysis of MTHFD2 protein expression after ATF4 knockdown in PC3 and DU145 cells. **(C)** ATF4 motif consensus sequence from JASPAR. The nucleotide sequences of the -1,494/-897 region in the human MTHFD2 gene and the candidate ATF4 binding sites (red text). **(D)** Dual-luciferase reporter assay to evaluate the effect of ATF4 on the activity of the WT-MTHFD2 promoter and its mutants. **(E)** ChIP assay to verify the binding of ATF4 to the MTHFD2 promoter in PC3 and DU145 cells. * *p* < 0.05; ** *p* < 0.01; *** *p*< 0.001.
